# Rapid Metagenomic Sequencing of Bronchoalveolar Lavage Fluid for Diagnosis of Infection in Patients With Hematologic Malignancies and Pulmonary Complications

**DOI:** 10.1016/j.chpulm.2025.100173

**Published:** 2025-05-30

**Authors:** Matthew K. Hensley, Khaled Sayed, Ghady Haidar, Xiaohong Wang, Panayiotis V. Benos, Sawa Ito, Annie Im, Emily Geramita, Warren Shlomchik, Barbara Methé, Charles Dela Cruz, Alison Morris, Georgios D. Kitsios

**Affiliations:** Department of Medicine, Division of Pulmonary, Allergy, Critical Care and Sleep Medicine, University of Pittsburgh, Pittsburgh, PA; Department of Electrical & Computer Engineering and Computer Science, University of New Haven, West Haven, CT; Department of Medicine, Division of Infectious Diseases, University of Pittsburgh, Pittsburgh, PA; Department of Epidemiology, College of Public Health and Health Professions and College of Medicine, University of Florida, Gainesville, FL; Center for Medicine and the Microbiome, University of Pittsburgh, Pittsburgh, PA; Department of Medicine, Division of Hematology and Oncology, University of Pittsburgh, Pittsburgh, PA

**Keywords:** immunocompromised, lower respiratory tract infection, metagenomics, pulmonary complications

## Abstract

**Background:**

Diagnosing pulmonary complications (PCs) in hematologic malignancies remains challenging due to insensitive conventional microbiologic testing (CMT) and overlapping clinical manifestations of infectious and noninfectious pulmonary complications. For these reasons, empirical antimicrobials and immunosuppression (eg, corticosteroids) are used for prolonged periods.

**Research Question:**

How does metagenomic sequencing of the lower respiratory tract compare with conventional microbiologic testing among patients with hematologic malignancy?

**Study Design and Methods:**

Prospective proof-of-concept cohort study of 30 adult inpatients with hematologic malignancies and PCs who underwent bronchoscopy for suspected lower respiratory tract infection.

**Results:**

CMT identified a pathogen via culture- or polymerase chain reaction-based testing in 53% of patients. 16S sequencing demonstrated 66.7% positive and 42.9% negative concordance with CMT, while also identifying additional plausible respiratory pathogens in 59.3% of patients. Nanopore demonstrated 6.7% positive and 87.5% negative concordance with CMT and identified additional plausible respiratory pathogens in 42.3% of patients.

**Interpretation:**

Culture-independent sequencing approaches had modest agreement with CMT when considering bacterial PCs and showed poor detection of fungal pathogens. Sequencing frequently identified additional plausible respiratory pathogens, and further validation is needed to determine if such detection represents clinically missed infections or nonpathogenic colonization.

**Take-Home Points**

**Study Question:** How does microbial sequencing (16S and Nanopore long DNA reads) aid in understanding the respiratory tract microbiome and potential diagnostic yield in patients with hematologic malignancy and pulmonary complications?

**Results:** Conventional microbiologic testing identified a pathogen in 53% of patients, with 16S sequencing showing 66.7% positive and 42.9% negative concordance and Nanopore demonstrating 6.7% positive and 87.5% negative concordance. Both sequencing methods identified additional plausible respiratory tract pathogens in nearly one-half of patients.

**Interpretation:** Culture-independent sequencing approaches had modest agreement with conventional testing when considering bacterial pneumonia and showed poor detection of fungal pathogens, while simultaneously detecting additional plausible pathogens not detected by culture-based methods. Integration of sequencing with established diagnostic algorithms for diagnosis of pulmonary complications among immunocompromised patients requires further refinement of techniques and established significance thresholds.

Pulmonary complications (PCs) in the setting of hematologic malignancies (HMs), including hematopoietic cell transplantation, are common and morbid. Up to 60% of patients develop infectious or noninfectious PCs, contributing to a mortality risk between 30% and 60%.^1^, ^2^, ^3^ Differentiating infectious entities (eg, fungal lower respiratory tract infection [LRTI]) from noninfectious PCs (eg, alveolar hemorrhage) is difficult due to overlapping manifestations, poor sensitivity of microbiologic cultures, limited specificity of antigen detection tests, and prolonged turnaround times of conventional microbiologic testing (CMT).^4^^,^^5^ Tailoring therapy is challenging because nearly 70% of patients with presumed LRTI lack identifiable pathogens,^6^^,^^7^ yet often receive extended courses of broad-spectrum empirical antimicrobials. Immunosuppression is frequently used empirically after antimicrobials among noninfectious PCs, which represent diagnoses of exclusion.^1^

There is a need for better diagnostics that can accurately and rapidly diagnose LRTI. We conducted the Rapid Metagenomic Sequencing Study (RaMSeS), a proof-of-concept observational cohort study to characterize microbial communities using microbial DNA sequencing of bronchoalveolar lavage (BAL) samples in PCs of different etiologies. To that end, we used 2 complementary sequencing methodologies: 16S ribosomal RNA gene sequencing (16S-Seq) and Nanopore metagenomics. 16S-Seq is a widely validated method that amplifies a conserved bacterial gene region to identify bacterial taxa in respiratory samples, offering a broad overview of bacterial diversity, but limited to bacterial identification. Illumina 16S-Seq is suited to large-scale, retrospective analyses due to its high-throughput capabilities, but lacks real-time reporting, making it more appropriate for epidemiologic studies.^8^ In contrast, Nanopore sequencing offers real-time, long-read sequencing that can capture entire DNA fragments, not just specific gene regions. This comprehensive approach enables detailed analysis of the entire microbial community, including bacteria, viruses, and fungi, and detection of potential antibiotic resistance markers. Nanopore’s ability to produce long reads and analyze DNA in real time means that species-level identification and resistance profiling can be achieved in clinically relevant turnaround times, as early as 6 to 8 hours, allowing immediate clinical decision-making.^9^, ^10^, ^11^

We performed sequencing analyses using both 16S-Seq and Nanopore metagenomics on aliquots of BAL fluid collected during the diagnostic workup of 30 patients with HMs and PCs. Our goal was to characterize these microbial communities through sequencing and to compare the potential diagnostic yield of sequencing approaches for identifying infectious PCs with that of CMT.

## Study Design and Methods

### Study Cohort

We prospectively enrolled adult (≥ 18 years of age) hospitalized patients with HM and a diagnosis of PCs (defined as hypoxemia or new/worsening radiographic abnormality) who underwent clinical diagnostic bronchoscopy from March 1, 2022, to October 30, 2022. One author (M. K. H.) obtained informed consent as part of University of Pittsburgh institutional review board-approved STUDY21110006:21-221. We collected at least 10 mL of BAL fluid for research use from the same lobe as clinical BAL. We recorded demographics, antimicrobial treatments, CMT results, and clinical outcomes up to 30 days after bronchoscopy.

### BAL Fluid Processing and Microbial DNA Sequencing Experiments

We kept BAL fluid on ice until same-day processing with centrifugation at 1,500 rpm for 5 minutes to separate pellets from supernatants. We divided pellets into 2 separate aliquots and performed human DNA depletion with a saponin-based method in the first aliquot,^10^ followed by genomic DNA extraction (human DNA depleted sample) for Nanopore metagenomic sequencing on the Mk1c MinION device (Oxford Nanopore Technologies). From the second aliquot, we performed DNA extraction (undepleted sample) for performance of amplicon 16S rRNA gene sequencing (Illumina MiSeq) for bacterial community profiling.^10^^,^^12^ We considered only samples that produced ≥ 50 microbial reads in each sequencing approach. From undepleted DNA samples, we also performed quantitative polymerase chain reaction of the V3 to V4 region of the 16S rRNA gene to obtain the number of gene copies per sample, as a surrogate for bacterial load.

### Clinical Consensus Definitions

We classified infectious PCs as follows: (1) bacterial LRTI was defined as CMT-positive based on established guidelines^13^ or CMT-negative (based on negative CMT but with consistent radiographic/clinical findings with LRTI and improvement with antibiotics); (2) viral LRTI was defined based on positive BAL or nasal polymerase chain reaction-based viral panel with a compatible clinical syndrome,^14^ whereas the specific case of cytomegalovirus pneumonitis was defined per established guidelines^15^; (3) fungal LRTI was defined based on the European Organisation for Research and Treatment of Cancer-Mycoses Study Group criteria^16^; (4) mixed LRTI was defined as having a combination of plausible pathogens from different kingdoms (eg, viral pneumonia with bacterial superinfection); and (5) noninfectious PCs were defined as PCs with negative CMT results and an alternative diagnosis identified either by biopsy or improvement with nonantimicrobial therapies (eg, diuretics for pulmonary edema, steroids for drug pneumonitis).

### Comparisons of Diagnostic Yield Between Sequencing-Based Approaches and CMT

For CMT-confirmed infectious PCs, we defined agreement (positive concordance) for 16S-Seq or Nanopore when each sequencing method detected a plausible causal respiratory pathogen among the top 3 abundant taxa and that pathogen was also detected by CMT (agreement at genus level for 16S-Seq for bacteria, and at species level for Nanopore). When sequencing revealed an additional pathogen different from the CMT-defined one, sequencing-identified pathogens were considered as additive diagnostic yield. For noninfectious PCs, we defined agreement (negative concordance) when sequencing did not reveal a plausible pathogen in agreement with the negative results of CMT.

For additional details on CMT, microorganism, and empirical antimicrobial definitions, see e-Appendix 1.

## Results

### Demographics and Clinical Outcomes

We enrolled 30 patients from March to October 2022. The median age was 66 years (interquartile range, 52-72), 12 (40.0%) were female, and 18 (60.0%) were neutropenic (absolute neutrophil count < 500 cells/μL) in the week before bronchoscopy. Thirteen (43.3%) had acute myeloid leukemia, and 10 (33.3%) had prior hematopoietic cell transplantation (Table 1). Clinical consensus classifications (groups) defined 22 patients (73.3%) as having infectious PCs (4 bacterial, 4 viral, 9 fungal, 5 mixed) and 8 patients (26.7%) as having noninfectious PCs (e-Table 1, Table 1). The median length of stay was 17 days (interquartile range, 4-39), 23 (76.7%) developed respiratory failure, 15 (50.0%) required ICU transfer, 10 (33.3%) required mechanical ventilation, and 10 (33.3%) died within 30 days of bronchoscopy.

**Table 1:** Demographics, Outcomes, and Sequencing Results by Clinical Consensus Group

Pulmonary Complications Clinical Consensus Category	Overall (N = 30)	Bacterial LRTI (n = 4)	Mixed LRTI (n = 5)	Viral LRTI (n = 4)	Fungal LRTI (n = 9)	Noninfectious PC (n = 8)
Demographics						
Age, y (Median, IQR)	66 (52-72)	58 (46-65)	72 (71-80)	63 (48-75)	67 (59-67)	58 (43-73)
Sex, female	12 (40)	1 (25)	2 (40)	1 (25)	7 (78)	1 (13)
Neutropenia, absolute neutrophil count < 500 in the week prior to bronchoscopu	18 (60)	3 (75)	1 (20)	3 (75)	7 (78)	4 (50)
Prior HCT	10 (33)	1 (25)	2 (40)	1 (25)	3 (33)	3 (38)
Hematologic malignancy type, AML	13 (43)	2 (50)	3 (60)	1 (25)	5 (56)	2 (25)
Hospitalization outcomes						
Length of stay (days, median, IQR)	17 (4-39)	11 (4-30)	14 (12-23)	20 (11-39)	19 (5-42)	18 (11-39)
Respiratory failure	23 (77)	3 (75)	4 (80)	3 (75)	5 (56)	3 (50)
ICU admission	15 (50)	3 (75)	2 (40)	3 (75)	3 (33)	4 (50)
Intubation	10 (33)	3 (75)	0 (0)	2 (50)	2 (22)	3 (38)
Death within 30 d of bronchoscopy	10 (33)	3 (75)	1 (20)	2 (50)	2 (22)	2 (25)
Total days of empirical gram-negative antibiotics	8 (3-13)	3 (1-9)	8 (7-13)	10 (5-12)	9 (5-14)	6 (1-9)
Total days of empirical gram-positive antibiotics	1 (0-2)	0 (0-1)	1 (0-1)	1 (0-5)	3 (0-5)	1 (0-2)
Total days of empirical antifungals	9 (0-41)	1 (0-8)	57 (10-100)	0 (0-6)	28 (16-104)	0 (0-15)
Sequencing results						
16S sequencing						
Positive concordance with CMT	4/6 (67)	3/4 (75)	1/2 (50)	NA	NA	NA
Negative concordance with CMT	3/7 (43)	NA	NA	NA	NA	3/7 (43)
Additive diagnostic yield (additional pathogen identification)	16/27 (59)	3/4 (75)	3/4 (75)	3/4 (75)	5/8 (63)	2/7 (29)
Nanopore sequencing						
Positive concordance with CMT	1/15 (7)	1/3 (33)	0/3 (0)	0/1 (0)	0/8 (0)	NA
Negative concordance with CMT	7/8 (88)	NA	NA	NA	NA	7/8 (88)
Additive diagnostic yield (additional pathogen identification)	11/26 (42)	2/3 (66)	2/3 (66)	2/4 (50)	4/8 (50)	1/8 (13)

Data are presented as median (interquartile range), No. (%), or total No./No. (%). AML = acute myeloid leukemia; CMT = conventional microbiologic testing; HCT = hematopoietic cell transplant (autologous, allogeneic, haploidentical); LRTI = lower respiratory tract infection; NA = not applicable; PC = pulmonary complication.

### Empirical Antimicrobial Therapy

Most patients (90%) were already receiving outpatient antibiotic treatment or prophylactic antibiotics before hospital admission. During their hospitalization and before bronchoscopy, all patients had received empirical broad-spectrum antibiotics (eg, piperacillin/tazobactam, cefepime, meropenem, vancomycin, caspofungin). The total duration of empirical antimicrobials was 8 (3-13 days; IQR), 1 (0-2 days; IQR), and 9 (0-41 days; IQR) median days of gram-negative, gram-positive, and antifungals, with no significant differences in empirical antimicrobial exposure between PCs groups (e-Fig 1, Table 1). Additionally, days of empirical treatments did not differ between those with and without neutropenia.

### Sequencing Results

**Figure 1:**
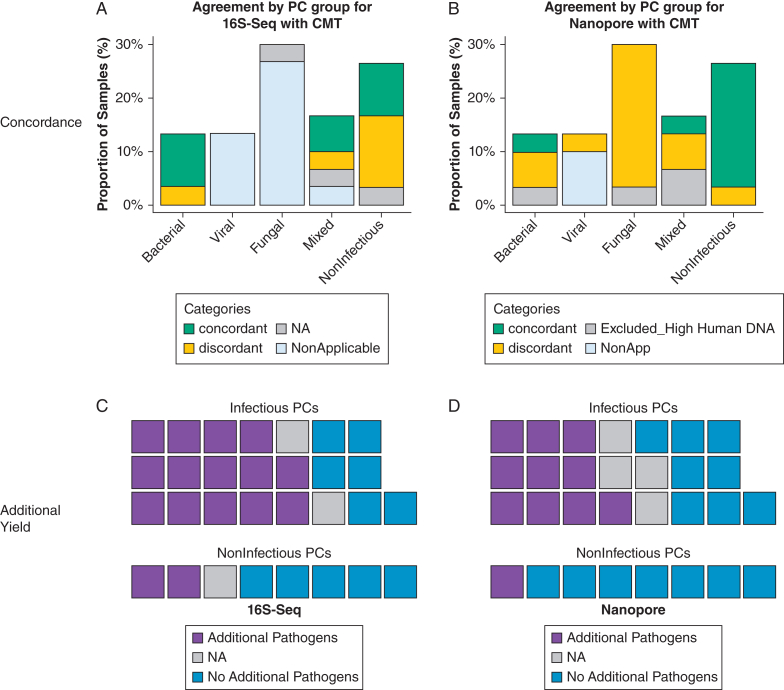
A-D, Agreement and additive yield of sequencing techniques with CMT. A, C, 16S-Seq results in comparison with CMT. B, D, Nanopore results in comparison with CMT. A, B, Stacked bar plots indicating concordance (green), discordance (yellow), exclusion due to high human DNA (gray), and not applicable (white). C, D, Waffle plots indicate additional pathogens (purple), not applicable (gray), or no additional pathogens (blue). 16S-Seq = 16S rRNA gene sequencing; CMT = conventional microbiologic testing; NA = not applicable; NonApp = not applicable; PC = pulmonary complication.

**Figure 2:**
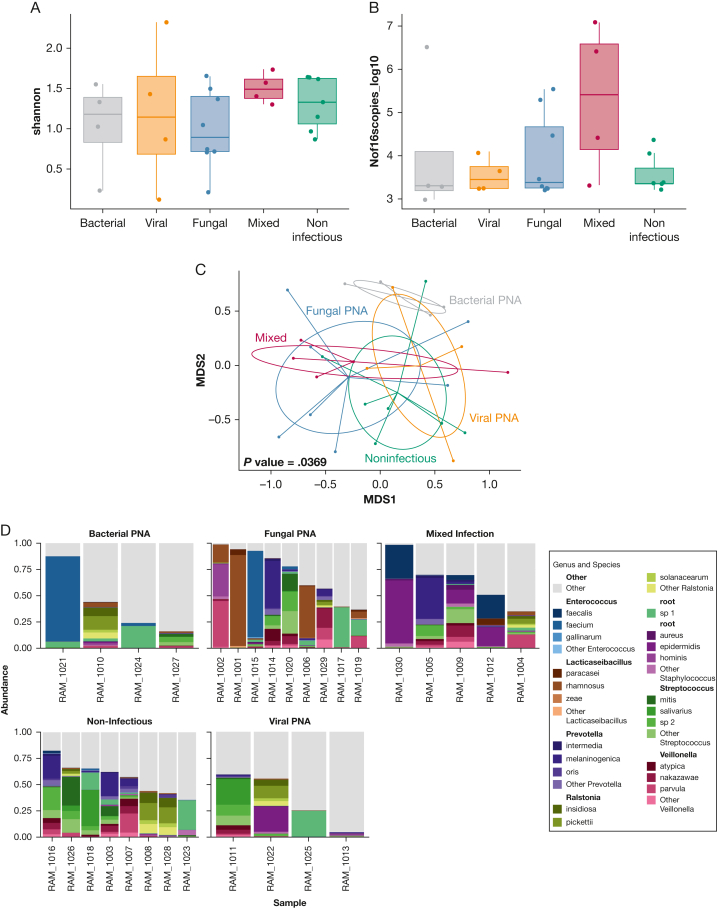
A-D, Sequencing results demonstrate similar diversity and microbial abundance. A, Alpha diversity (community diversity) measured by the Shannon index did not differ between groups. B, Total microbial burden by 16S sequencing did not differ between groups. C, Multidimensional scale plots showing significant different beta diversity between groups. D, Species level data by diagnostic group using Nanopore sequencing. PNA = pneumonia.

CMT identified plausible lower respiratory tract pathogens in 16 patients (53%) (e-Table 1). By Nanopore sequencing, human DNA reads accounted for 74.5% of total sequencing output. Of the 30 cases, 4 (13.3%) had ≥ 99% human DNA by Nanopore and were excluded from further comparisons (e-Table 1, Fig 1). For 16S-Seq, 3 samples (10.0%) were not available due to operational reasons. We found no difference in bacterial load by 16S quantitative polymerase chain reaction or alpha diversity by 16S-Seq between clinical PC groups. Beta-diversity was significantly different between groups, with bacterial LRTIs appearing more dissimilar in composition compared with the other groups (*P* = .04) (Fig 2). We did not find any significant difference in the proportions of microbial vs human DNA reads by Nanopore sequencing between PC categories (*P* = .68), and in total amounts of microbial or human DNA reads (e-Figs 2, 3). We hypothesized that patients with neutropenia may have lower amounts of human DNA and higher amounts of microbial DNA in BAL samples, but we found no significant differences between patients with and without neutropenia (e-Fig 4).

In total, CMT identified a pathogen via culture- or polymerase chain reaction-based testing in 53% of patients. 16S-Seq demonstrated 66.7% positive concordance, demonstrated 42.9% negative concordance with CMT, and identified additional plausible respiratory pathogens in 59.3% of patients. Nanopore demonstrated 6.7% positive concordance and 87.5% negative concordance with CMT, and it identified additional plausible respiratory pathogens in 42.3% of patients (Fig 1, Table 1).

### Comparisons of Sequencing Technologies With CMT by Clinical Classifications

#### Bacterial LRTI

Of the 22 (73.3%) infectious LRTIs, 4 (13.3%) were classified as culture-positive bacterial (*Pseudomonas aeruginosa*: n = 3, *Stenotrophomonas* species: n = 1 by BAL culture). Of these, 16S-Seq identified the causal pathogen among the top 3 abundant taxa in all 3 cases of *P aeruginosa*, but not in the *Stenotrophomonas* pneumonia case, for an overall positive concordance of 75.0% (3 of 4). Additional plausible respiratory pathogenic taxa were identified in 3 cases (*Staphylococcus* species in patient 10, *Enterococcus* species in patient 21, and *Enterococcus* species in patient 24). Nanopore sequencing identified the plausible causal pathogen identified by conventional testing in 1 case (*P aeruginosa*), for a positive concordance of 33.3% (1 of 3, case 10 excluded for large amount of human DNA reads). Additional clinically relevant pathogens were detected in 2 cases (*Enterococcus faecium* and *Escherichia coli* in patients 21 and 24).

#### Viral LRTI

Four individuals (13.3%) were diagnosed with isolated viral LRTIs (SARS-CoV-2 in case 22, parainfluenza in case 25, rhino-enterovirus in case 11, and cytomegalovirus in case 13). In these patients, 16S-Seq suggested additional plausible clinically relevant bacterial taxa not identified by CMT in 3 of 4 patients (75%) (*Pseudomonas* species in case 13, *Pseudomonas* species and *Staphylococcus* species in cases 22 and 25). Nanopore sequencing identified cytomegalovirus DNA in case 13 but below the significance threshold (relative abundance > 1%), therefore showing a percent agreement of 0%. Nanopore sequencing identified additional clinically relevant bacterial pathogens in 2 of 4 patients (50.0%) (*Haemophilus parainfluenzae* in case 11, *E coli* in case 25). Of note, case 25 had both BAL and blood fungal cultures positive for *Candida guilliermondii*. This case had the highest number of *Candida* reads (identified as *Candida dubliniensis*) in BAL fluid by Nanopore sequencing (e-Table 1).

#### Fungal LRTI

Nine patients (30.0%) had fungal LRTIs: 1 proven (*Aspergillus* species on biopsy), 7 probable (positive *Lomentospora prolificans* on culture: n = 1, galactomannan > 0.5 optical density index or elevated beta-D-glucan: n = 3, *Pneumocystis* polymerase chain reaction positive: n = 3), and 1 possible (neutropenia with consistent imaging and otherwise negative CMT workup). Of these 9 patients, 16S-Seq identified additional clinically relevant bacterial taxa in 5 of 8 patients (62.5%) (*Burkholderia* species in case 1, *Staphylococcus* species in case 2, *Actinomyces* species in case 14, *Enterococcus* species in cases 15 and 17). Nanopore sequencing did not detect any abundant pathogenic fungal species in these 9 fungal LRTIs. However, *C dubliniensis* reads were detected in 7 of 9 cases (77.8%), with no significant differences in number of reads compared with other groups. In the possible fungal LRTI (case 2) where CMT was also negative, Nanopore did not identify fungal or plausible bacterial pathogens. Nanopore suggested additional clinically relevant bacterial pathogens in 4 of 8 patients (50%) (*E faecium* in cases 15 and 20; *E coli* in cases 15, 17, 19, and 20; *Streptococcus pneumoniae* in case 20; *Staphylococcus aureus* in case 15) (e-Table 1, Fig 1).

#### Mixed LRTI

Five patients (16.7%) had mixed infectious LRTIs (SARS-CoV-2 and probable invasive fungal infection (IFI): n = 1, *Actinomyces* species and possible IFI: n = 1, 1 *S aureus* and probable IFI: n = 1, culture-negative bacterial LRTI and probable IFI: n = 1, SARS-CoV-2 and *Pneumocystis jirovecii*: n = 1). Of the 2 patients that had bacterial pathogen involvement, 16S-Seq identified the plausible causal bacterial taxon (*Staphylococcus* species) in case 9, but missed the CMT-defined causal pathogen in the other (*Actinomyces* species in case 5), for a percent agreement of 50%. 16S-Seq also suggested additional clinically relevant bacterial taxa not identified by CMT in 3 of 4 patients (75%) (*Stenotrophomonas* species in case 4, *Actinomyces* species in case 9, *Staphylococcus* species in cases 9 and 12, *Enterococcus* species in case 12). Two cases were excluded from Nanopore sequencing due to large amounts of human DNA. Nanopore sequencing did not detect the CMT-defined bacterial pathogens in the 2 cases of mixed LRTIs with bacterial coinfection, for a percent agreement of 0%, but suggested additional clinically relevant pathogens in 2 of 3 patients (66.7%) (*Enterococcus faecalis* in cases 12 and 30, *S aureus* in case 30) (e-Table 1, Fig 1).

#### Noninfectious PCs

Of the 8 patients (26.7%) classified as having noninfectious PCs, 16S-Seq identified additional clinically relevant bacterial taxa in 2 of 7 patients (28.6%) (*Staphylococcus* species in case 8, *Enterococcus* species in case 23) (e-Table 1, Fig 1). Nanopore sequencing identified additional clinically relevant pathogens in 1 of 8 patients (12.5%) (*E coli* and *E faecium* in case 18) (e-Table 1, Fig 1).

## Discussion

In this study of immunocompromised patients with HM, 16S-Seq showed modest agreement, whereas Nanopore showed poor agreement with CMT. We also show no significant differences between microbial diversity or abundance across clinical PC groups, suggesting that clinical diagnoses do not capture differences in underlying microbial communities. Sequencing technologies did identify additional clinically relevant pathogens in more than one-half of patients, but interpretation of those results is difficult given the lack of abundance thresholds. In patients with fungal PCs, defined according to the combined European Organisation for Research and Treatment of Cancer-Mycoses Study Group definitions, Nanopore metagenomic sequencing demonstrated poor positive concordance, yet suggested additional clinically relevant bacterial pathogens that CMT may have failed to identify. In patients with clinically diagnosed viral LRTIs, sequencing indicated the possibility of high rates of bacterial superinfections that CMT may have missed. Among noninfectious etiologies, 16S-Seq identified additional plausible clinically relevant pathogens in a minority of noninfectious PCs, whereas Nanopore sequencing showed good agreement (negative concordance) with CMT in > 75% of patients.

Our results build on prior work in immunocompromised hosts.^7^^,^^17^^,^^18^ In a review of metagenomic sequencing investigations among patients with HMs, CMT demonstrated wide variability (1%-87%) in detecting pathogens, whereas metagenomic sequencing increased detection with a range of 24% to 100%.^18^ In a cohort with nearly one-half the patients being immunocompromised, 16S-sequencing of multiple tissue (bone, abscess, skin) and fluid (BAL) samples was concordant with conventional testing in > 80% of patients.^17^ Another study investigated metagenomic Illumina sequencing of BAL samples among 30 immunocompromised adults, where CMT identified a pathogen in one-third of patients.^7^ The addition of sequencing demonstrated an additional 9% diagnostic yield relative to CMT alone. Our results do not show significant agreement between CMT and metagenomic sequencing, contradictory to prior literature, for several possible reasons. Specifically, we observed poor agreement between Nanopore metagenomic sequencing and CMT in this cohort, both for bacteria and fungi. All patients received antimicrobials before bronchoscopy which may have inhibited the ex vivo growth of certain pathogens, leading to discrepancies between CMT and sequencing results. Detection of several pathogens by sequencing alone suggests that these organisms may have been present during the PC diagnosis, but potentially no longer viable at the time of bronchoscopy. It remains uncertain whether continuing antibiotics when a pathogen is detected only by sequencing—without corresponding growth in cultures—would be clinically appropriate because these organisms may be nonviable or inhibited rather than actively contributing to the infection.

High amounts of human DNA in a few samples may have adversely impacted results for Nanopore, which can comprehensively sequence long DNA molecules with low risk of bias, offering taxonomic resolution at species level, including fungi and DNA viruses, which can expand the diagnostic yield beyond bacteria captured by 16S-Seq. However, high amounts of human DNA in samples can adversely impact microbial sequencing yield. Specifically, for fungal LRTIs, Nanopore did not detect abundant fungal DNA from molds; however, it identified *Candida* species and other yeasts. This may be attributed to the low fungal DNA biomass often observed in BAL fluid samples, as demonstrated in other studies comparing quantitative polymerase chain reaction yield for *Aspergillus* species and *Pneumocystis* species, where bronchial aspirate showed higher fungal loads than BAL fluid in detecting these pathogens.^19^ Additionally, our human DNA-depletion protocol, which relies on intact cellular walls for lysis via saponin,^20^ might have inadvertently removed fungal DNA, especially from molds with thick cell walls. Conversely, the bead-based DNA extraction methods we used may have been more effective at extracting DNA extraction from yeasts, but less so from molds.^21^^,^^22^ Finally, some of the IFI cases may have been misclassified as infectious PCs due to reliance on antigen detection (eg, beta-D-glucan). Future research should consider alternative human DNA depletion and microbial DNA extraction methods, specifically for molds.^23^

Although we hypothesized that patients with neutropenia would exhibit higher microbial burden due to impaired clearance, we showed similar burdens across groups. This finding suggests that other immune cells (eg, resident macrophages, lymphocytes) may help modulate microbial burden in the absence of neutrophils. The human DNA detected may primarily arise from airway or alveolar epithelial cells and appear independent of neutrophil status; however, the cellular origin of sequenced human DNA remains uncertain. Advanced epigenomic analyses could clarify these origins and their relationship with microbial burden but were beyond the scope of this study. Further research is needed to better understand the interplay between microbial burden, immune response, and tissue injury in patients with and without neutropenia.

Nanopore and 16S-Seq identified additional clinically relevant pathogens in 12.5% to 28.6% of noninfectious PCs that were not detected by CMT. This finding aligns with prior studies indicating that nearly one-half of patients with noninfectious PCs (eg, idiopathic pneumonia syndrome) harbor plausible pathogens detectable by sequencing but missed by CMT,^24^ suggesting potential misclassification when relying solely on CMT. Although microbial DNA sequencing shows promise in enhancing diagnostic capabilities and influencing treatment decisions,^9^ it has yet to meet the analytical and clinical standards of validated diagnostic tests. The discordance observed between 16S-Seq and Nanopore results in identifying additional pathogens (e-Table 1) underscores the technologic differences and limitations inherent to these approaches. For example, 16S-Seq detects only short bacterial DNA amplicons, which can introduce primer biases that favor certain taxa, whereas Nanopore sequencing analyzes long-read DNA molecules in samples that have been subjected to host DNA depletion protocols, which may have inadvertently altered the microbial community. Furthermore, differences in taxonomic classification databases (ie, SILVA for 16S-Seq, national center for biotechnology information for Nanopore) and operational rank abundance thresholds (eg, such as our use of the top 3 taxa, optimized for non-HM populations) add complexity to result interpretation.^10^^,^^25^ All patients received antimicrobials before bronchoscopy, which may have inhibited the ex vivo growth of certain pathogens, leading to discrepancies between CMT and sequencing results. Detection of several pathogens by sequencing alone suggests that these organisms may have been present during the PC diagnosis, but potentially no longer viable at the time of bronchoscopy. It remains uncertain whether continuing antibiotics when a pathogen is detected only by sequencing—without corresponding growth in cultures—would be clinically appropriate because these organisms may be nonviable or inhibited rather than actively contributing to the infection.

The absence of established abundance thresholds to differentiate true infections from colonization or microbial background further complicates sequencing result interpretation, particularly when organisms of uncertain pathogenicity are detected in noninfectious PCs. Bridging sequencing outputs with traditional microbiological standards requires critical methodologic advances. For instance, correlating sequencing results with bacterial concentrations (eg, 10^4^ colony forming units in BAL cultures^13^) established as diagnostic thresholds for pneumonia could clarify how read counts correspond to clinically relevant microbial loads. This is especially pertinent for organisms (eg, *E faecium*) whose role in respiratory infections is debated yet often prompts treatment in immunocompromised patients.^26^ *Enterococcus* species have been implicated in approximately 2% of pneumonia cases in critically ill patients,^27^ but more frequent detection by sequencing will generate challenges in interpretation. Therefore, interpretation of sequencing profiles would benefit from concurrent profiling of lower respiratory tract host responses to help distinguish contextually active pathogens from colonizers. Until sequencing evolves into a validated diagnostic tool, integrating clinical, microbiological, and sequencing data remains essential for optimizing its utility in managing complex LRTI cases.

Limitations of this study include its observational design and small sample size, which restrict generalizability and statistical power. The diagnostic reference standard for LRTI, relying on culture- or antigen-based tests, has limited sensitivity and specificity.^11^ To address time-related diagnostic limitations, we used a retrospective consensus approach among 3 physicians to integrate clinical and microbiological data. Although our approach represents pragmatic physician diagnostic thinking on available CMT results, it introduces potential subjectivity. Antimicrobial treatment before bronchoscopy likely reduced the yield of CMT disproportionately compared with sequencing, complicating the interpretation of discrepancies between methods in the absence of a reference standard diagnostic technology. Moreover, we reported only the most abundant taxa, leaving lower-abundance pathogens unexplored to avoid overinterpretation; however, they may still hold clinical relevance. The absence of standardized methods to interpret sequencing data (eg, established correlations with microbial loads, clinical outcomes) further complicates the application of these findings to clinical practice.

## Interpretation

Culture-independent microbial DNA sequencing approaches demonstrated limited overall agreement with CMT in our study. However, these methods may provide added diagnostic yield in immunocompromised patients by identifying bacterial pathogens that CMT misses. Despite their promise, current metagenomic approaches show significant limitations in detecting molds, emphasizing the need for further methodologic refinement to enhance their diagnostic utility for fungal LRTIs. Additionally, the detection of plausible pathogens in cases classified as noninfectious PCs suggests potential misclassification by CMT, or the possibility that some noninfectious PCs may have an infectious trigger that was previously unrecognized. These findings underscore the need for ongoing research to integrate sequencing technologies with established diagnostic paradigms, refine interpretation criteria, and improve their application in managing complex PCs in immunocompromised patients with HM.

## Funding/Support

G. D. K. was supported by the 10.13039/100000050NHLBI [Grant R03 HL162655] and the 10.13039/100002590American Lung Association COVID-19 Respiratory Virus Research Award. G. H. was supported by the 10.13039/100000060National Institute of Allergy and Infectious Diseases [Grant K23AI154546]. M. K. H. was supported by the 10.13039/100007454Shadyside Hospital Foundation. P. V. B. was supported by the NHLBI [Grant R01 HL159805].

## Financial/Nonfinancial Disclosures

The authors have reported to *CHEST Pulmonary* the following: G. D. K. has received research funding from Karius and Genentech, unrelated to this work. G. D. K. and A. M. have received research funding from Pfizer, unrelated to this work. G. H. is a recipient of research grants from Allovir, Karius, Regeneron, AstraZeneca, and the Cystic Fibrosis Foundation; serves on the scientific advisory boards of Karius, AstraZeneca, and SNIPR BIOME; and has received honoraria from the International AIDS Society, MDOutlook, ARLG, MAD-ID/ID connect, PeerView Institute for Medical Education, and PRIME Inc, unrelated to this work. W. S. is a stockholder and compensated consultant for Bluesphere Bio and holds stock options in Orca Bio. None declared (M. K. H., K. S., X. Z., P. V. B., S. I., A. I., E. G., B. M., C. D. C.).

## Supplementary Material

e-Online Data
