# Supplementary material for: Rapid Metagenomic Sequencing of Bronchoalveolar Lavage Fluid for Diagnosis of Infection in Patients With Hematologic Malignancies and Pulmonary Complications
Source: CHEST Pulm. Author manuscript; Available in PMC 2026 Feb 6. (PMC12875665; doi:10.1016/j.chpulm.2025.100173)
Supplement: Supplementary file 1 [file NIHMS2126044-supplement-1.docx]

**e-Supplemental Methods**

Conventional Microbiologic Testing and Spectrum of Antimicrobial Definitions

CMT obtained at the discretion of the clinical team included bacterial or fungal cultures (blood, BALF, sputum, or other sites), serologies, antigen-based biomarkers (e.g., fungal biomarkers (serum, urine, BALF), *Legionella*, other), and PCR tests (respiratory viruses, *Pneumocystis jirovecii*). We classified antimicrobials into antibiotics with Gram-negative or Gram-positive bacteria coverage or antifungals (e-Table 2). 

Microorganism Definitions

We defined microorganisms detected by sequencing as plausible respiratory pathogens based on expert knowledge regarding their likelihood as cause of LRTI in immunosuppressed hosts (e-Table 3). Conversely, we classified microorganisms as commensals or of unclear clinical relevance, when such organisms have been classified as members of the healthy lung microbiome or are not implicated in causing LRTI based on prior work and expertise^10,12^. CMT-defined pathogens were included if identified on culture-based testing, biopsy proven (e.g., *Aspergillus* on lung biopsy) or PCR-based results (e.g. SARS-CoV-2). Positive galactomannan or beta-D-glucan were not sufficient for pathogen identification, but did aid in consensus diagnostic classification. 

For both 16S-Seq and Nanopore sequencing, we reported the top 3 most abundant taxa in each BALF sample at the genus level based on previous proof-of-concept work showing that the causal pathogen in CMT-confirmed bacterial pneumonias is among the top 3 abundant taxa in cases of severe bacterial pneumonia among mechanically ventilated immunocompetent hosts^10^. To further delineate the causal organisms by Nanopore sequencing at the species level, we considered bacterial or fungal species as plausible pathogens if their relative abundance was ≥1% (e.g., species DNA reads/total microbial reads) and if human DNA represented less than 99% of total classifiable reads. We chose this conservative threshold to mitigate reporting of false-positive results from species of extremely low abundance.

Total Days of Empiric Antimicrobials

We measured total days of empiric antimicrobial therapy for each patient, defined as untargeted antimicrobials started by the clinical team until either tailoring of antimicrobials to CMT-identified pathogens or when terminated by clinicians by the time they considered a PC to be non-infectious. If CMT did not reveal an infectious etiology, but the PC was determined to be infectious by consensus review, we considered total duration of antimicrobials as empiric. The total days of therapy (DOT) were calculated by adding the individual days each patient was on an individual antimicrobial (e.g., 2 days of vancomycin plus 3 days of piperacillin/tazobactam equals 5 DOT). We excluded routine prophylactic antimicrobials^17^. We tracked duration of antimicrobials based on chart review, follow-up notes, or refills for up to one-year post-hospitalization in the outpatient setting for fungal LRTI participants, as they often require prolonged duration of antifungals without clear guidance on when to conclude treatment^18^. 

Data Processing and Analyses: 

We assessed alpha diversity, which measures the biodiversity within individual samples by considering both richness (number of different taxa) and evenness (distribution of taxa) within the community, using the Shannon index. For respiratory communities, higher alpha diversity has been associated with greater community fitness and overall respiratory health^19^. To evaluate beta diversity—compositional differences or similarities between microbial communities across samples—we used Manhattan distances, which we analyzed via permutation analysis of variance and visualized through principal coordinates analysis. We performed analyses in R with the *vegan* and *mia* packages. The Kruskal-Wallis test was used to assess differences in median days of therapy via STATA 17.0. Results were considered significant if p<0.05. 

| e-Table 1. Patient History, CMT Results, Diagnosis, and Sequencing Results^^^. |  |  |  |  |  |  |
| --- | --- | --- | --- | --- | --- | --- |
| Patient ID | **Clinical History** | **Total Days of Empiric Antimicrobials** | **Conventional Microbiologic Testing (abnormal results) *** | **Final Clinical Diagnosis** | **16S rRNA gene sequencing (Total reads with top 3 bacterial taxa and corresponding reads and relative abundance if >1%)** | **Nanopore sequencing results (Total classifiable reads, top 3 taxa, plausible respiratory pathogens with relative abundance >1%) ^** |
| Bacterial LRTI |  |  |  |  |  |  |
| 10 | 59-year-old with myelodysplastic syndrome transformed to acute myeloid leukemia admitted with respiratory failure and ground glass opacities. | 31 | Respiratory: initial BAL cultures no growth. Later repeat bronchoscopy bacterial culture grew *Pseudomonas aeruginosa*. Blood: BD-glucan positive (340 pg/ml) | Pseudomonas pneumonia | Total 8,830 ***Staphylococcus*** **1,882 (21.3%)** ***Pseudomonas*** **863 (9.8%)** *Prevotella* 782 | Total 265,025 Human DNA 264,069 *Ralstonia* 270 *Herbaspirillium* 210 *Lacticaseibacillus* 43 *excluded from analysis due to large amount of human DNA |
| 21 | 70-year-old with acute myeloid leukemia and recent COVID-19 admitted with neutropenic fever and new infiltrates concerning for pneumonia. | 4 | Respiratory: BAL cultures grew *Pseudomonas* *aeruginosa* Blood: Blood cultures grew *Pseudomonas* *aeruginosa* BD glucan 231 pg/mL | *Pseudomonas* pneumonia | Total 9,890 ***Pseudomonas*** **7,175 (72.5%)** ***Enterococcus*** **2,416 (24.4%)** *Acinetobacter* 71 | Total 39,131 Human DNA 34,610 *Enterococcus*  3634 *Escherichia* 202 *Pseudomonas 100* ***Enterococcus faecium*** **(3,618; 9.2%)** |
| 24 | 35-year-old with chronic myeloid leukemia in blast crisis, *Enterococcus* bacteremia, *Stenotrophomonas* bacteremia, prior fungal pneumonia admitted with neutropenic fever and bacteremia and developed respiratory failure. | 0 | Respiratory: Initial BAL no growth, later repeat BAL culture grew *Stenotrophomonas* *maltophila*. Blood: culture positive for *Enterococcus* *faecium* repeatedly, *Stenotrophomonas* *maltophila* | *Stenotrophomonas* pneumonia, *Enterococcal* bacteremia | Total 9,984 ***Enterococcus*** **9,923 (99.4%)** *Lactobacillus* 15 *Afipia* 14 | Total 20,712 Human DNA 456 *Pseudomonas* 7,114 *Escherichia* 3,236 *Cutibacterium* 618 ***Escherichia coli*** **(2,956; 14.27%)** ***Enterococcus faecium*** **(588, 2.83%)** |
| 27 | 57-year-old with T-cell prolymphocytic leukemia status post hematopoietic cell transplant, prior histoplasmosis, persistently positive for COVID-19, admitted with fever, hypoxia, tree-in-bud opacities. | 2 | Nasal: PCR positive for SARS-CoV-2 (persistently positive) Respiratory: BAL cultures positive for *Pseudomonas* aeruginosa, fungal culture with non-cryptococcal yeast, galactomannan 1.8 | *Pseudomonas* *aeruginosa* pneumonia | Total 9,673 ***Pseudomonas*** **8,504 (87.9%)** *Prevotella* 624 *Veilonella* 205 | Total 926,724 Human DNA 584,416 *Pseudomonas* 260,497 *Streptococcus* 40,177 *Veilonella* 7,516 ***Pseudomonas aeruginosa*** **(237,187; 25.59%)** |
| Viral LRTI |  |  |  |  |  |  |
| 11 | 74-year-old with diffuse large B-cell lymphoma s/p chimeric antigen therapy admitted with cough and ground glass opacities. | 14 | Respiratory – viral panel positive for *Rhino-enterovirus* Blood – initial cultures no growth. Later repeat blood cultures positive for *Pseudomonas* *aeruginosa* and *Enterococcus* *faecalis*. Urine: culture positive for *Pseudomonas* *aeruginosa*. | Rhino-enterovirus pneumonia Later in hospitalization developed Pseudomonal/Enterococcal bacteremia | Total 8,649 *Veilonella* 2,702 *Streptococcus* 2,260 (26.1%) *Prevotella* 1,752 | Total 35,248 Human DNA 446 *Streptococcus* 15,640 *Veilonella* 3,463 *Schaalia* 3,100 ***Haemophilus parainfluenzae*** **(1,571, 4.5%)** |
| 13 | 52-year-old with multiple myeloma status post hematopoietic cell transplant, bispecific engager therapy, recent spinal cord compression, readmitted with fever and ground glass opacities | 29 | Respiratory: no abnormal results Blood: *Cytomegalovirus* PCR positive (2,303,902 IU/mL) | Cytomegalovirus pneumonia/viremia | Total 9,966 ***Pseudomonas*** **6,337 (63.6%)** *Caulobacteraceae* 2,034 *Pantoea* 1,280 | Total 3,823 Human DNA 3,314 *Ralstonia* 153 *Herbaspirillium* 127 *Lacticaseibacillus* 22 |
| 22 | 44-year-old with follicular lymphoma admitted with fever and new infiltrates concerning for pneumonia | 11 | Respiratory: viral panel positive for *SARS-CoV-2* from BAL, negative from nasal PCR | COVID-19 pneumonia | Total 9,692 ***Pseudomonas*** **3,832 (39.5%)** ***Staphylococcus*** **3,340 (34.5%)** *Pantoea* 927 | Total 80,166 Human DNA 77,247 *Staphylococcus* 731 *Ralstonia* 696 *Herbaspirillium* 529 |
| 25 | 75-year-old with chronic lymphocytic leukemia/small lymphocytic lymphoma admitted for chimeric antigen therapy, developed fever and parainfluenza, later recurrent fever, and ground glass opacities. | 0 | Nasal: positive for *Parainfluenza* type 4 Respiratory: Fungal culture positive for *Candida* *guilliermondii*, galactomannan 0.09 Blood: culture positive for *Candida* *guilliermondii*, BD-glucan 454 pg/mL | *Parainfluenza* pneumonia, *Candidemia* | Total 9,931 *Lactobacillus* 6,068 ***Staphylococcus*** **2,932 (29.5%)** ***Pseudomonas*** **360 (3.6%)** | Total 103,334 Human DNA 36,336 *Escherichia* 11,873 *Candida* 3,847 *Kluyveromyces* 2,533 ***Escherichia coli*** **(9,810; 9.49%)** |
| Fungal LRTI |  |  |  |  |  |  |
| 1 | 67-year-old with new diagnosis of acute myeloid leukemia admitted for induction chemotherapy, developed respiratory failure with ground glass opacities | 44 | Respiratory: Positive *Pneumocystis jirovecii* PCR | Probable IFI, *Pneumocystis jirovecii* | Total 9,636 ***Burkholderia*** **6,585 (68.3%)** *Lactobacillus* 2,797 *Veilonella* 148 | Total 109,860 Human DNA 4,083 *Lacticaseibacillus* 97,980 *Lautropia* *mirabilis* 5,502 *Ralstonia* 261 |
| 2 | 67-year-old with new acute myeloid leukemia admitted for chemotherapy, developed pulmonary  consolidation and neutropenic fever | 59 | No growth or abnormal results | Possible IFI | Total 9,813 *Veilonella* 7,477 *Lactobacillus* 1,420 ***Staphylococcus*** **760 (7.7%)** | Total 223,333 Human DNA 56,591 *Veilonella* 76,796 *Staphylococcus* 54,783 *Lacticaseibacillus* 28,282 |
| 6 | 74-year-old with multiple myeloma s/p hematopoietic cell transplant, recent COVID-19, admitted with fever, respiratory failure, concern for pneumonia | 14 | Respiratory: positive cytology for possible yeast and pseudohyphae, BAL galactomannan positive (1.26) | Probable IFI, (COVID-19 associated pulmonary aspergillosis) | Total 9582 *Lactobacillus* 6,608 *Rothia* 1,552 *Streptococcus* 443 (4.6%) | Total 450,072 Human DNA 446,000 *Lacticaseibacillus* 2,015 *Candida* 775 *Rothia* 165 *excluded from analysis due to large amount of human DNA |
| 14 | 61-year-old with diffuse large B-cell lymphoma status post chimeric antigen therapy admitted with chest pain and left lower lobe air space disease. | 1 (cefepime) | Respiratory: FNA positive for *Aspergillus* species (not *fumigatus*) Blood: BD glucan positive (>500 pg/mL) | Proven IFI (*Aspergillus*) | Total 9,739 *Prevotella* 4,466 *Veilonella* 2,226 ***Actinomyces*** **1,063  (10.9%)** | Total 1,254,278 Human DNA 23,236 *Prevotella* 654,970 *Veilonella* 217,767 *Streptococcus* 160,515 |
| 15 | 68-year-old admitted with new acute myeloid leukemia, developed respiratory failure | 36 | Respiratory: fungal cultures positive for *Lomentospora* (*Scedosporium*) *prolificans*, galactomannan negative (0.04) | Probable IFI (*Scedosporium*) | Total 9,700 ***Enterococcus*** **7,231 (74.5%)** *Veilonella* 2,127 *Methylobacterium* 89 | Total 84,527 Human DNA 66,519 *Enterococcus* 14,790 *Cutibacterium* 410 *Streptococcus* 397 ***Enterococcus faecium*** **(14,575; 17.24%)** |
| 17 | 66-year-old with acute myeloid leukemia status post hematopoietic cell transplant admitted with neutropenic fever with concern for pneumonia | 33 | Respiratory: BAL cultures earlier in hospital stay grew *Aspergillus* *fumigatus*, repeat cultures were without growth. Galactomannan 8.14 and persistently positive on repeat sampling. RVP positive for *Rhino-enterovirus* Blood: initial cultures negative, repeat cultures later in hospital stay positive for *Enterococcus* *faecium*. BD-glucan >500 pg/mL | *Aspergillus* pneumonia (probable IFI), *Enterococcal* bacteremia | Total 9,975 ***Enterococcus*** **9,949 (99.7%)** *Mitochondria* 12 *Pseudomonas* 7 | Total 71,239 Human DNA 26,462 *Escherichia* 10,138 *Acinetobacter* 447 *Salmonella* 443 ***Escherichia coli*** **(8,525; 11.97%)** |
| 19 | 49-year-old with thyroid cancer and new acute myeloid leukemia admitted with neutropenic fever and ground glass opacities. | 123 | Respiratory: fungal culture showed non-cryptococcal yeast, galactomannan 0.30 Blood – serum BD glucan 196 pg/mL | Probable IFI | Total 9,334 *Veillonella* 4,480 *Rothia* 2,975 *Lactobacillus* 714 | Total 41,422 Human DNA 36,781 *Rothia* 982 *Escherichia* 558 *Veilonella* 524 ***Escherichia coli*** **(506, 1.22%)** |
| 20 | 58-year-old with breast cancer, myelodysplastic syndrome status post hematopoietic cell transplant admitted with neutropenic fever and found to have cavitary pulmonary nodules. | 106 | Respiratory: Initial workup negative. Later BAL galactomannan 10.69 Blood:BD glucan 126 pg/mL | Probable IFI | Total 9,690 *Veilonella* 4,095 *Prevotella* 1,725 *Rothia* 1,274 | Total 103,332 Human DNA 5,510 *Streptococcus*  55,529 *Veilonella* 12,894 *Schaalia* 6,520 ***Streptococcus pneumoniae*** **(3,684; 3.57%)** ***Enterococcus faecium*** **(2,378; 2.3%)** |
| 29 | 35-year-old with chronic myeloid leukemia transformed to acute myeloid leukemia admitted for chemotherapy, later developed neutropenic fever and cough with left sided infiltrates concerning for pneumonia. | 277 | Blood: BD glucan 166 pg/mL | Possible IFI | Unavailable | Total 200,565 Human DNA 181,638 *Veilonella* 7,001 *Prevotella* 2,005 *Actinomyces* 1,751 |
| Mixed LRTI |  |  |  |  |  |  |
| 4 | 80-year-old with chronic myeloid leukemia and history of fungal pneumonia admitted with respiratory failure and multi-focal pneumonia. | 24 | Respiratory: viral panel positive for *SARS-CoV-2* Respiratory: Galactomannan positive (0.62) | COVID-19 pneumonia, probable IFI | Total 9,034 *Neisseria* 2,241 ***Stenotrophomonas*** **1,073 (11.9%)** *Prevotella* 831 | Total 433,860 Human DNA 432,592 *Leptotrichia* 381 *Veilonella* 161 *Ralstonia* 136 *excluded due to large amount of human DNA |
| 5 | 72-year-old admitted with new acute myeloid leukemia, developed neutropenic fever and concern for pneumonia. | 79 | Respiratory: *Actinomadura nitrigenes* on BAL AFB culture. | *Actinomyces* pneumonia, possible IFI | Total 9,532 *Prevotella* 5,030 *Veilonella* 2,806 *Granulicatella* 357 | Total 325,644 Human DNA 64,201 *Prevotella* 118,809 *Streptococcus* 34,632 *Leptotrichia* 26,516 |
| 9 | 65-year-old with acute myeloid leukemia s/p haploidentical hematopoietic cell transplant, graft versus host disease of skin/gut/lung p/w fever, chills, dyspnea, and ground glass opacities. | 111 | Respiratory: positive BAL bacterial culture for *Staphylococcus* *aureus*. Blood: BD-glucan >500 | Staphylococcal  pneumonia, probable IFI | Total 9,021 *Veilonella* 5,218 *Prevotella* 1,123 *Campylobacter* 1,123 ***Actinomyces*** **605 (6.7%)** ***Staphylococcus*** **520 (5.8%)** | Total 231,340 Human DNA 231,205 *Veilonella* 31 *Staphylococcus* 27 *Streptococcus* 20 *excluded due to large amount of human DNA |
| 12 | 80-year-old admitted with new acute myeloid leukemia and concern for pneumonia | 109 | Respiratory: fungal culture positive for non-cryptococcal yeast Blood: positive serum BD glucan (122 pg/mL) | Culture-negative bacterial pneumonia, probable IFI | Total 8,681 ***Staphylococcus*** **6,036 (69.5%)** ***Enterococcus*** **2,238 (25.8%)** *Lactobacillus* 302 | Total 9,790 Human DNA 5,461 *Enterococcus* 946 *Candida* 941 *Staphylococcus* 800 ***Enterococcus faecalis*** **(921, 9.4%)** |
| 30 | 63-year-old with myelodysplastic syndrome, aplastic anemia status post hematopoietic cell transplant, recent COVID-19 s/p monoclonal antibody admitted with hypoxic respiratory failure and ground glass opacities. | 8 | Respiratory: BAL negative for *SARS-CoV-2. Pneumocystis* PCR detected, cytology negative | COVID-19 pneumonia, Probable *Pneumocystis* | Unavailable | Total 207,641 Human DNA 129,542 *Staphylococcus* 49,757 *Enterococcus* 24,884 *Streptococcus* 760 ***Enterococcus faecalis*** **(22,289; 10.73%)** |
| Non-infectious |  |  |  |  |  |  |
| 3 | 32-year-old with porphyria s/p hematopoietic cell transplant, liver/kidney transplant, who developed post-transplant lymphoproliferative disorder status post chimeric antigen therapy and admitted with cough, chest pain with diffuse ground glass opacities. | 7 | Nasal: viral panel positive for *Adenovirus* Respiratory – viral PCR negative | Non-infectious – surgical lung biopsy later showed graft versus host disease | Total 8,129 *Prevotella* 4,169 *Veilonella* 2,283 *Alloprevotella* 341 | Total 14,478 Human DNA 14,239 *Prevotella* 61 *Streptococcus* 44 *Veilonella* 28 |
| 7 | 77-year-old admitted for new acute myeloid leukemia, developed respiratory failure, fever, and ground glass opacities | 37 | Respiratory: no abnormal results. Fine needle aspirate showed malignant cells. | Non-infectious – leukemic infiltrates | Total 9576 *Prevotella* 3790 *Veilonella* 3227 *Rothia* 313 | Total 850 Human DNA 598 *Veilonella* 88 *Prevotella* 37 *Cutibacterium* 33 |
| 8 | 46-year-old with Hodgkin’s lymphoma and cryptogenic organizing pneumonia admitted with respiratory failure and diffuse ground glass opacities | 23 | Respiratory: viral panel positive for *rhino-enterovirus* Blood: serum BD glucan positive (484 pg/ml), *Cytomegalovirus* Viremia (75,621 IU/mL) | Cryptogenic organizing pneumonia – non-infectious, possible CMV reactivation | Total 9,952 ***Staphylococcus*** **3,863 (38.8%)** *Streptococcus* 2,246 (22.6%) *Rubellimicrobium* 1,647 | Total 123,382 Human DNA 120,888 *Ralstonia* 918 *Herbaspirillium* 628 *Phyllobacterium* 126 |
| 16 | 70-year-old with cardiomyopathy recent diagnosis of acute myeloid leukemia admitted with respiratory failure | 6 | No abnormal results | Non-infectious – pulmonary edema | Total 9,402 *Prevotella* 4,947 *Veilonella* 2,398 *Rothia* 814 | Total 505,561 Human DNA 127,317 *Prevotella* 113,418 *Streptococcus* 110,522 *Veilonella* 65,531 |
| 18 | 60-year-old with large B-cell lymphoma, colon cancer, s/p hematopoietic cell transplant admitted with increasing dyspnea and cough. | 0 | Respiratory: BAL cytology showed malignant cells. FNA showed airway lymphomatous involvement | Disease progression of lymphoma | Total 8,623 *Veilonella* 2,609 *Prevotella* 2,236 *Streptococcus* 1,312 (15.2%) | Total 210,410 Human DNA 28,608 *Streptococcus* 73,374 *Escherichia* 25,696 *Veilonella* 3,692 ***Escherichia coli*** **(22,834; 10.85%)** ***Enterococcus faecium*** **(2,215; 1.1%)** |
| 23 | 40-year-old with chronic myeloid leukemia status post hematopoietic cell transplant, relapsed disease now with blasts, fungal pneumonia, nocardia esophagitis, admitted with acute respiratory failure and ground glass opacities | 107 | Blood – cultures positive for *Staphylococcus* *haemolyticus*, BD-glucan 183 pg/mL Surgical lung biopsy showed pulmonary alveolar proteinosis without infectious pathogens | Pulmonary alveolar proteinosis secondary to underlying leukemia | Total 8,779 ***Enterococcus*** **2,867 (32.7%)** *Corynebacterium* 1,201 *Cloacibacterium* 985 | Total 63,659 Human DNA 62,015 *Ralstonia* 530 *Herbaspirillium* 376 *Staphylococcus* 106 |
| 26 | 55-year-old with chronic myeloid leukemia admitted with blast crisis. Developed dyspnea, respiratory failure, ground glass opacities and pleural effusions. | 3 | No abnormal results. | Drug pneumonitis from dasatinib | Total 8,128 *Gemella* 2,840 *Rothia* 2,325 ***Streptococcus*** **1,335 (16.4%)** | Total 169,272 Human DNA 164,437 *Streptococcus* 2,566 *Haemophilus* 478 *Rothia* 389 |
| 28 | 75-year-old with blastic plasmacytoid dendritic cell neoplasm admitted with splenomegaly and acute kidney injury. Later developed respiratory failure and diffuse bilateral infiltrates. | 11 | No abnormal results | Non-infectious - Diffuse alveolar hemorrhage | Unavailable | Total 153,181 Human DNA 150,389 *Ralstonia* 944 *Herbaspirillium* 699 *Phyllobacterium* 138 |
| *Unless otherwise specified, respiratory cultures for acid-fast bacilli, fungal, and bacterial pathogens were no growth or normal flora, cytology negative for infectious pathogens, biomarkers negative for fungal infection, viral PCR negative for COVID-19, influenza, respiratory syncytial virus, adenovirus, rhino/enterovirus, blood cultures were no growth, Pneumocystis jirovecii PCR not detected. Abbreviations: IFI (invasive fungal infection) ^Conventional microbiological testing was directed by the clinical care team and included combinations of blood cultures, BAL cultures for bacterial, viral, and fungal pathogens, BAL cytologic analysis for pathogens, BAL PCR testing for *Pneumocystis jirovecii*, BAL and serum galactomannan (fungal cell wall marker), serum β-d-glucan (fungal cell wall marker), and BAL or nasopharyngeal swab quantitative PCR testing (qPCR) for respiratory viruses. Viral pneumonia was diagnosed by positive qPCR testing on respiratory viral panels, whereas IFI was defined according to EORTC/MSG guidelines^13^. *P.* *jirovecii* was defined as probable IFI based on the EORTC/MSG guidelines^13^. |  |  |  |  |  |  |

| **e-Figure 1: Proportion of Microbial vs. Human DNA Reads from Nanopore Sequencing** Legend: (A, top) individual subjects graph demonstrating a high proportion of human DNA reads in 4 cases leading to exclusion from analysis; (B, bottom). No significant differences in proportion of human DNA reads were found between PC categories. **e-Figure 2: Nanopore Sequencing Shows No Differences in Human DNA or Microbial Burden** Legend: (A, top) Total human DNA reads by diagnostic category; (B, bottom) Total microbial DNA reads stratified by diagnostic category **e-Figure 3: No Difference in Human DNA, Microbial Burden, or Proportion of Microbes Between Neutropenic and Non-Neutropenic Patients Using Nanopore Sequencing.** Legend: A) total human DNA reads stratified by neutropenia; B) total microbial DNA reads stratified by neutropenia; C) proportion of microbial DNA reads stratified by neutropenia. **e-Table 2. List of antimicrobials considered empiric by category*** |  |  |
| --- | --- | --- |
| Empiric gram-negative antimicrobials | Empiric gram-positive antimicrobials | Empiric antifungals |
| Piperacillin/tazobactam | Vancomycin | Caspofungin |
| Cefepime | Linezolid | Isavuconazonium |
| Ceftolozane/tazobactam | Daptomycin | Amphotericin |
| Ceftazidime/avibactam | Ceftaroline | Voriconazole |
| Aztreonam |  | Posaconazole |
| Meropenem with or without vaborbactam |  | Bactrim (Pneumocystis) |
| Imipenem with or without relebactam |  | Clindamycin/Primaquine (Pneumocystis) |
| *Antimicrobials are not mutually exclusive (Piperacillin can be used to treat gram-positive pathogens) but are specified as gram-negative empiric coverage in our study given their clinical use in the hematologic malignancy population. |  |  |

| **e-Table 3. Definition of Microorganisms** |  |
| --- | --- |
| Plausible Clinically Relevant Pathogens | Organisms of Unclear Significance or Commensals |
| *Staphylococcus aureus* | *Prevotella* |
| *Pseudomonas aeruginosa* | *Ralstonia* |
| *Enterococcus faecium* | *Lacticaseibacillus* |
| *Enterococcus faecalis* | *Cutibacterium* |
| *Haemophilus parainfluenzae* | *Afipia* |
| *Escherichia coli* | *Lactobacillus* |
| *Burkholderia spp (16s)* | *Veilonella* |
| *Actinomyces spp (16s)* | *Schaalia* |
| *Streptococcus pneumoniae* | *Pantoea* |
| *Neisseria spp (16s)* | *Herbaspirillium* |
| *Salmonella spp (16s)* | *Caulobacteraceae* |
| *Campylobacter (16s)* | *Candida* |
| *Corynebacterium (16s)* | *Lautropia* |
|  | *Rothia* |
|  | *Methylobacterium* |
|  | *Leptotrichia* |
|  | *Granulicatella* |
|  | *Alloprevotella* |
|  | *Phyllobacterium* |
|  | *Rubellimicrobium* |
|  | *Cloacibacterium* |
|  | *Gemella* |
|  | *Streptococcus spp (non-pneumoniae)* |

**e-Figure 4**. **No Difference in Duration of** **Empiric Antimicrobial Therapies by Diagnostic Group**

Legend: Median total days of therapy (DOT) by PC Group for A) gram negative antimicrobials; B) gram positive antimicrobials; and C) antifungals. Refer to e-Table 2 for antimicrobial definitions.
